# Stent expansion evaluated by optical coherence tomography and subsequent outcomes

**DOI:** 10.1038/s41598-023-30717-6

**Published:** 2023-03-07

**Authors:** Bom Lee, Teklay Gebrehaweria Baraki, Byung Gyu Kim, Yong-Joon Lee, Seung-Jun Lee, Sung-Jin Hong, Chul-Min Ahn, Dong-Ho Shin, Byeong-Keuk Kim, Young-Guk Ko, Donghoon Choi, Myeong-Ki Hong, Yangsoo Jang, Jung-Sun Kim

**Affiliations:** 1 Division of Cardiology, Department of Internal Medicine, Severance Cardiovascular Hospital, Yonsei University College of Medicine, 03722 Yonsei-ro 50-1, Seodaemun-gu, Seoul, South Korea; 2 Division of Cardiology, Department of Internal Medicine, Sanggye Paik Hospital, Inje University College of Medicine, Seoul, South Korea; 3 Division of Cardiology, Department of Internal Medicine, CHA Bundang Medical Center, CHA University, Seongnam, South Korea

**Keywords:** Cardiology, Interventional cardiology

## Abstract

Regarding stent expansion indices, previous optical coherence tomography (OCT) studies have shown minimal stent area (MSA) to be most predictive of adverse events. We sought to evaluate the impact of various stent expansion and apposition indices by post-stent OCT on clinical outcomes and find OCT-defined optimal stent implantation criteria. A total of 1071 patients with 1123 native coronary artery lesions treated with new-generation drug-eluting stents with OCT guidance and final post-stent OCT analysis were included. Several stent expansion indices (MSA, MSA/average reference lumen area, MSA/distal reference lumen area, mean stent expansion, and stent expansion by linear model [stent volume/adaptive reference lumen volume]) were evaluated for their association with device-oriented clinical endpoints (DoCE) including cardiac death, target vessel-related myocardial infarction (MI) or stent thrombosis, and target lesion revascularization. MSA was negatively correlated with the risk of DoCE (hazard ratio [HR] 0.80 [0.68‒0.94]). However, stent expansion by linear model representing the overall volumetric stent expansion was associated with greater risk of DoCE (HR 1.02 [1.00‒1.04]). As categorical criteria, MSA < 5.0 mm^2^ (HR 3.90 [1.99‒7.65]), MSA/distal reference lumen area < 90% (HR 2.16 [1.12‒4.19]), and stent expansion by linear model ≥ 65.0% (HR 1.95 [1.03‒3.89]) were independently associated with DoCE. This OCT study highlights the importance of sufficient stent expansion to achieve adequate, absolute, and relative MSA criteria for improving clinical outcome. It also emphasises that overall volumetric excessive stent expansion may have detrimental effects.

## Introduction

Although drug-eluting stents (DES) have dramatically reduced the incidence of repeat revascularization, post-stent complications resulting from suboptimal stent expansion including restenosis and stent thrombosis still exist^1–3^. Adequate stent expansion has been recognized as an important aspect for stent optimization to reduce incidence of its failure^4^. Stent expansion has been described as the minimal stent area (MSA), either as an absolute expansion or compared with the predefined reference lumen area (relative expansion). Greater absolute stent expansion has been associated with better long-term stent patency and MSA of less than 4.5 or 5.4 mm^2^ on optical coherence tomography (OCT) in non-left main lesion is an independent predictor of subsequent events^5–7^. However, achievable MSA is limited when dealing with small vessels and cut-offs may result in undersized stents in large vessels. Regarding the relative stent expansion, it is recommended by an expert committee of the European Association of Percutaneous Coronary Intervention to achieve > 80% of MSA divided by average reference lumen area ^4^; however, the criteria for relative stent expansion have not been well elucidated. In addition, several relative stent expansion indices have been proposed considering reference vessel size and tapering ^8–10^; however, further investigations for their clinical impact is needed. Therefore, the present study aimed to evaluate the predictive value of several stent expansion indices assessed by post-stent OCT for long-term device-oriented clinical endpoints (DoCE) and determine OCT-defined optimal stent expansion criteria after new-generation DES implantation.

## Methods

The data that support the findings of this study are available from the corresponding author upon reasonable request.

### Study population and design

**Figure 1:**
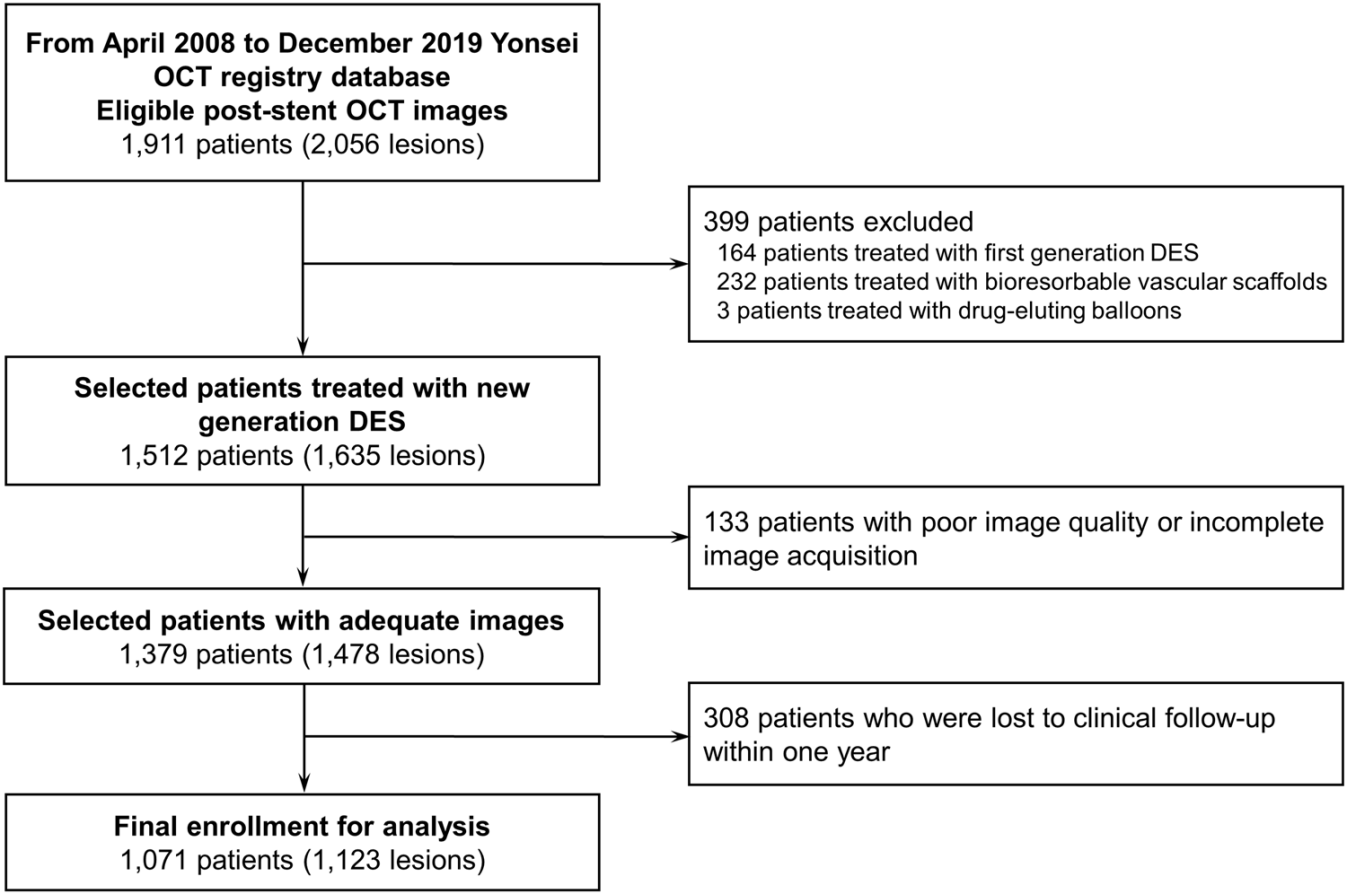
Study flow. Study population at each step and reasons for exclusion are described in detail. *DES* drug eluting stent, *OCT* optical coherence tomography.

The Yonsei OCT registry for evaluation of efficacy and safety of coronary stenting (NCT02099162) is a prospective, observational registry to evaluate the coronary anatomy, appropriateness of coronary stents during percutaneous coronary intervention (PCI), strut coverage at follow-up, and clinical outcomes after PCI^11^. OCT was conducted before or after PCI for de novo lesions, at follow-up angiography, or during PCI for the in-stent restenotic lesions according to the operator’s decision without randomization. For this study, we considered enrolment of subjects who received PCI for de novo lesions with new-generation DES and post-stent OCT examination immediately after PCI. The selection of stent size and length were left to operators’ discretion based on quantitative measurements of reference vessel size and lesion length by OCT. Stent deployment under OCT guidance also performed based on operators’ discretion without specific recommendation or guidelines for stent optimization target, but further optimization and repeat OCT was performed in case of suboptimal post-stent OCT image due to flow-limiting edge dissection, severe malapposition, or stent underexpansion. The final OCT images were evaluated in the present study. The study flow is provided in Fig. 1. From April 2008 to December 2019, 1911 patients (with 2,056 lesions) who underwent PCI for de novo lesions with post-stent OCT images were identified. A total of 399 patients treated with first generation DES implants (164 patients), bioresorbable vascular scaffolds (232 patients), and drug-eluting balloons (3 patients) were excluded. Other causes of exclusion of 133 patients were poor image quality, incomplete image acquisition of entire stent length or reference lumen, and no information about stent. A total of 308 patients who were lost to clinical follow-up within one year were excluded. Finally, a total of 1071 patients (with 1123 lesions) with post-stent OCT images after new-generation DES implantation were included in the present analysis. DES were selected by operator at the time of procedure and each one of them was implanted according to current standard techniques. Details of procedure and list of new-generation DES are given in “Supplementary Data”. This registry was approved by institutional review board of Severance Hospital, and all participants provided written informed consent. All methods were performed in accordance with the relevant guidelines and regulations.

### OCT measurements and definitions

Post-stent OCT images were acquired by a frequency-domain C7-XR OCT system (LightLabImaging Inc., St. Jude Medical, St. Paul, Minnesota) according to a previously described technique^12^. Images were analyzed by three independent analysts using certified off-line software (Qlvus, Medis Medical Imaging System, Leiden, The Netherlands) at a core laboratory (Cardiovascular Research Centre, Seoul, Korea). Cross-sectional OCT images were analyzed at every 1 mm interval; stent and lumen cross-sectional areas were measured. The volumes of each were calculated with Simpson’s rule^13^.

**Figure 2:**
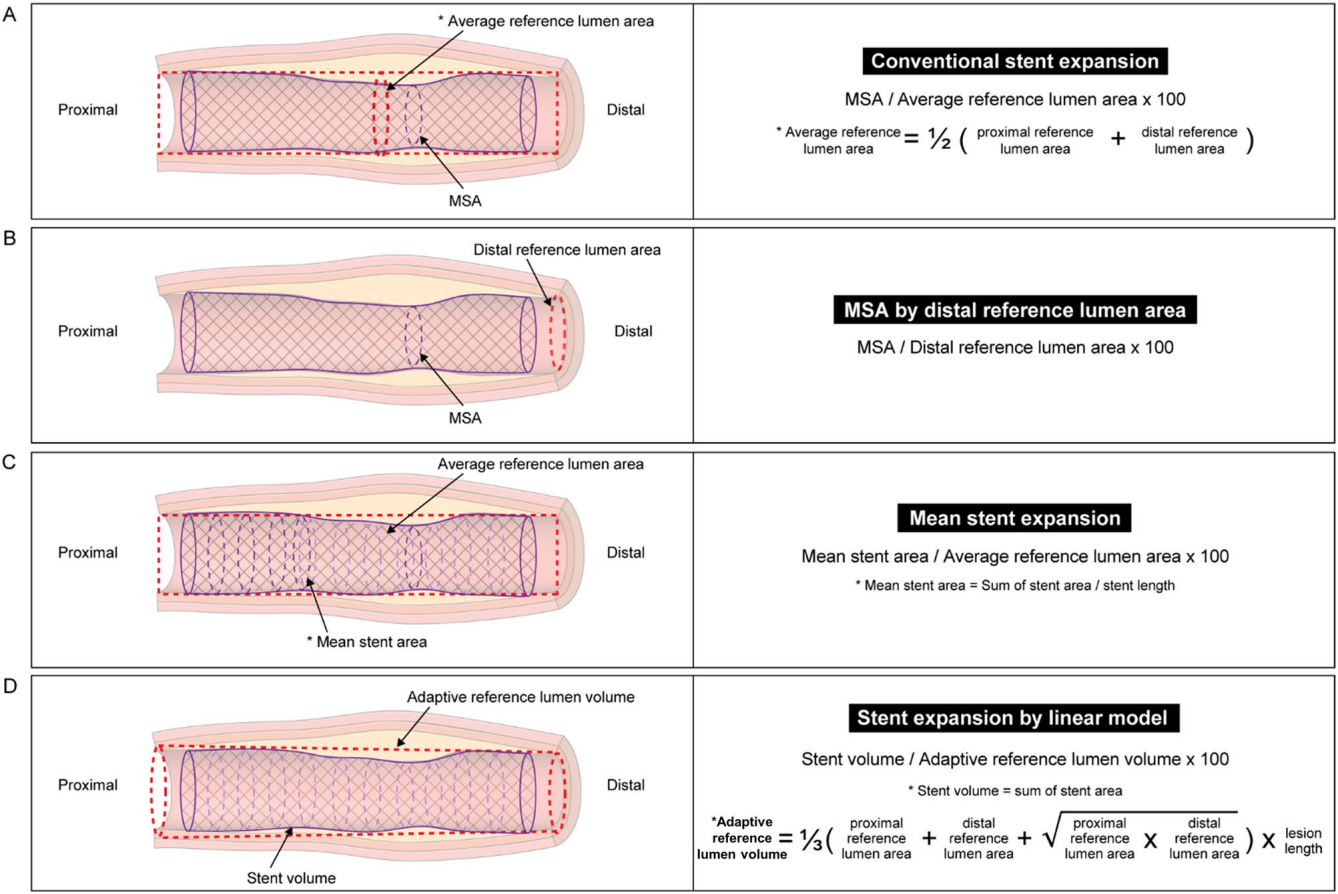
Stent expansion indices. (**A**) Conventional stent expansion, (**B**) MSA by distal reference lumen area, (**C**) mean stent expansion, and (**D**) volumetric stent expansion by linear model. *MSA* minimal stent area.

**Figure 3:**
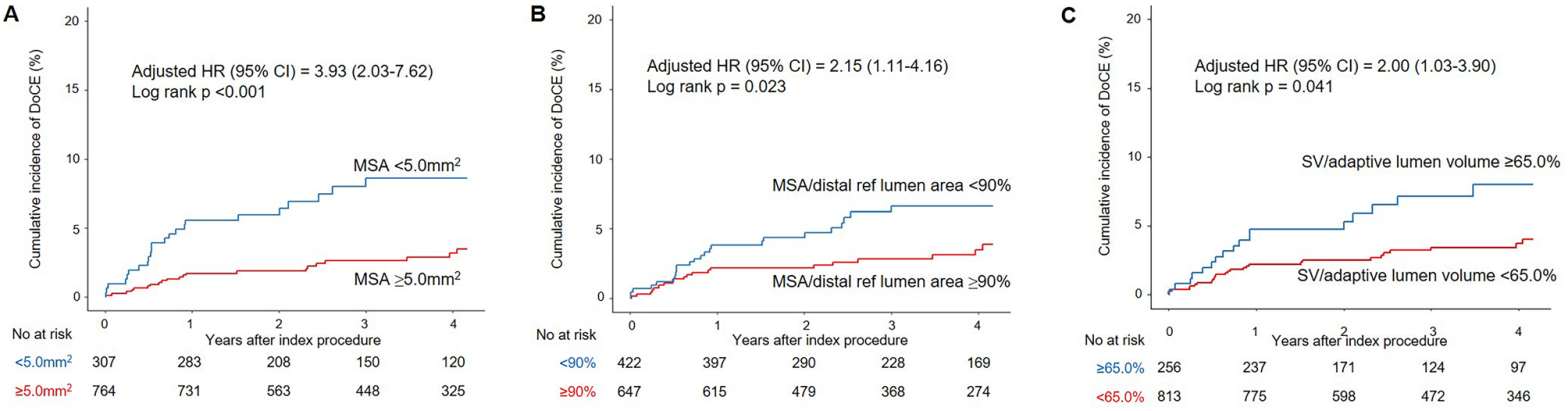
Kaplan–Meier curves according to OCT-defined suboptimal stent expansion criteria. Kaplan–Meier estimates for DoCE according to (**A**) MSA (< 5.0 mm^2^ versus ≥ 5.0 mm^2^), (**B**) MSA/distal reference lumen area (< 90% versus ≥ 90%), and (**C**) SV/adaptive reference volume (≥ 65.0% versus < 65.0%) Adjusted covariates include age, male sex, hypertension, diabetes, current smoker, chronic kidney disease, myocardial infarction, statin use at discharge, stent diameter, and total stent length. *CI* confidence interval, *DoCE* device-oriented clinical end point, *HR* hazard ratio, *MSA* minimal stent area, *OCT* optical coherence tomography, *SV* stent volume.

**Figure 4:**
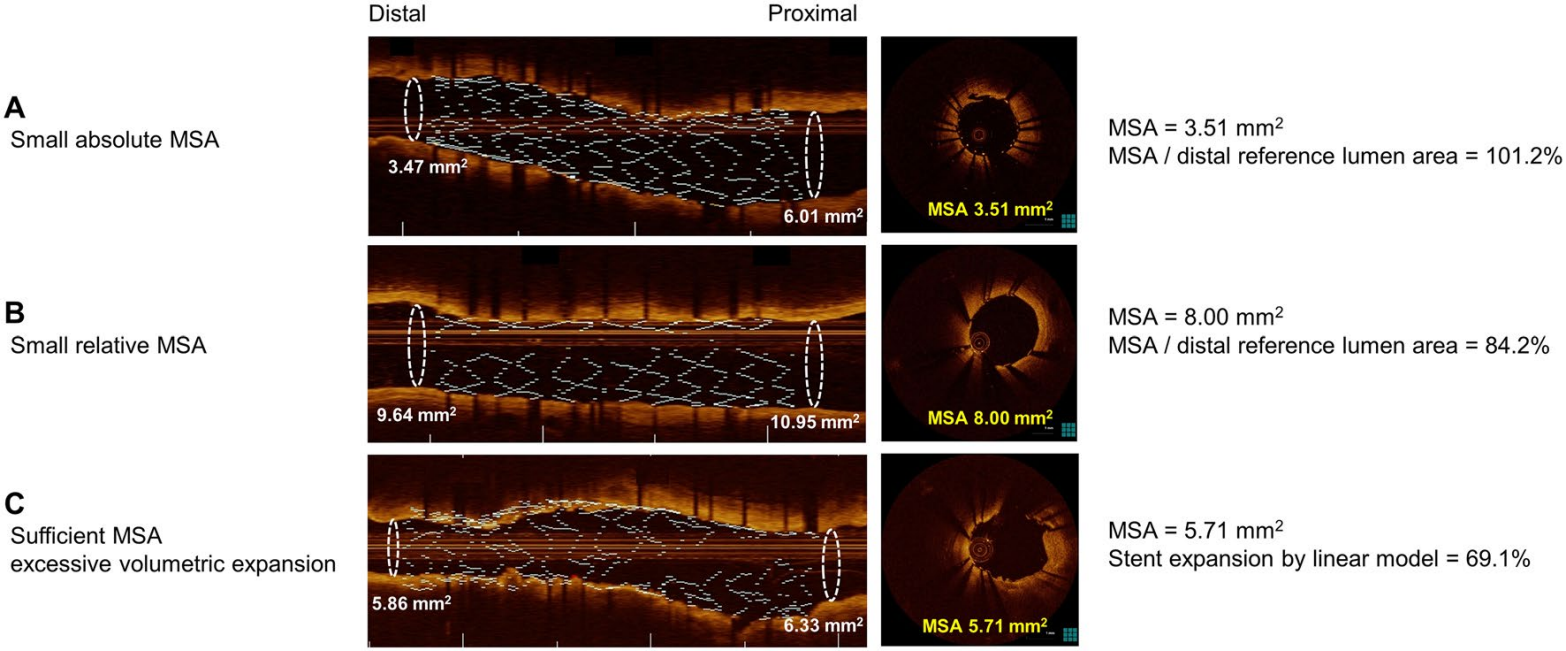
Representative OCT images of suboptimal stent expansion resulting in adverse outcomes. (**A**) MSA is small (< 5.0 mm^2^), resulting in TLR. (**B**) MSA is larger than 5.0 mm^2^ but the ratio of MSA by distal reference lumen area is less than 90%, resulting in TLR. (**C**) MSA is larger than 5.0 mm^2^ and volumetric stent expansion is over 65.0% of adaptive reference vessel volume, resulting in TLR. *DoCE* device-oriented clinical end point, *MSA* minimal stent area, *OCT* optical coherence tomography, *TLR* target lesion revascularization.

The following indices of stent expansion were investigated: (1) MSA; (2) conventional stent expansion: MSA/average reference lumen area (average of proximal and distal reference lumen area) (Fig. 2A); (3) MSA by distal reference lumen area: MSA/distal reference lumen area (Fig. 2B); (4) mean stent expansion: mean stent area (sum of stent area/analyzed stent length)/average reference lumen area^8^ (Fig. 2C); and (5) stent expansion by linear model: stent volume/adaptive reference lumen volume (Fig. 2D). The concept of volumetric stent expansion by linear model was referred to ILUMIEN I substudy ^9^, which used an ideal lumen area by creating an ideal lumen profile along the stent area in consideration of vessel tapering. Adaptive reference lumen volume was obtained by volume of a hypothetical frustum of a cone shape using a linear interpolation method between the proximal and distal references, and was calculated as $$\begin{aligned} & 1{/}3 \times \left( {{\text{proximal}}\;{\text{reference}}\;{\text{lumen}}\;{\text{area}} + {\text{distal}}\;{\text{reference}}\;{\text{lumen}}\;{\text{area}} + } \right. \\ & \left. {\sqrt {{\text{proximal}}\;{\text{reference}}\;{\text{lumen}}\;{\text{area}} \times {\text{distal}}\;{\text{reference}}\;{\text{lumen}}\;{\text{area}}} } \right) \times {\text{lesion}}\;{\text{length}} \\ \end{aligned}$$. Some previously proposed suboptimal stent expansion criteria were also evaluated: conventional stent expansion < 90% ^14^, conventional stent expansion < 80% ^4,7^, MSA by distal reference lumen area < 90% ^10^, and MSA < 5.0 mm^2^ ^5,15^.

### Clinical outcomes

DoCE included cardiac death, target vessel-related myocardial infarction (MI) or stent thrombosis, and target lesion revascularization (TLR). A post-hoc analysis was done for DoCE according to Academic Research Consortium^16^. Cardiac death was considered in case of an immediate cardiac cause or lack of evidence of a non-cardiac one. MI was defined by cardiac biomarker elevation with at least one value above the 99th percentile of the upper reference limit, with concomitant ischaemic symptoms or electrocardiographic findings indicative of ischaemia unrelated to an interventional procedure. TLR was defined as any repeat PCI of the target lesion, bypass surgery of the target vessel performed for restenosis, or other target lesion complications. Stent thrombosis was defined as definite or probable stent thrombosis. Clinical follow-up was performed periodically at six-month intervals either by a clinical visit or a telephonic interview. Clinical follow-up was conducted for up to four years and at least one-year follow-up has been completed for all patients enrolled in this study.

### Statistical analysis

Continuous variables are expressed as mean ± standard deviation, and categorical variables are expressed as numbers and frequencies. Group comparison were performed using the Mann–Whitney U, Pearson’s chi-squared, Fisher’s exact, or Student *t* test, as indicated. To determine the predictive value of each stent expansion indices and criteria for clinical outcomes, univariate and multivariate analyses using a Cox proportional hazards model was performed; age, sex, hypertension, diabetes, current smoker, chronic kidney disease, acute myocardial infarction, statin use at discharge, mean stent diameter, and total stent length were entered into the model. Harrell’s C-index was used to determine the predictability of the DoCE using volumetric stent expansion index^17^. Maxstat, a maximal chi-square method^18^ in R 3.6.0 was used to determine best cutting points for stent expansion by linear model to predict DoCE. The predictive performance of threshold of stent expansion by linear model was internally validated using the algorithm by Harrell et al. for bootstrap optimism correction using 1000 bootstrapped resampling^17^. It was examined whether Harrell’s C-index for the threshold of stent expansion by linear model was reproducible without much difference during the bootstrap procedure. Log-rank and Kaplan–Meier tests were used to compare incidence of DoCE according to the stent expansion criteria. Analyses of baseline demographics and OCT parameters were reported using available data without imputation for missing data, given the low rate of missing data (< 3%). There were no missing data regarding clinical follow-up. All tests were two-sided, and P < 0.05 was considered statistically significant. These analyses were performed using R Statistical Software (version 3.6.0; R Foundation for Statistical Computing, Vienna, Austria).

## Results

### Clinical and lesion characteristics

A total of 44 patients developed DoCE during the follow-up period (median 41.9 [interquartile range 22.3–50.0] months). There were no significant differences in baseline clinical characteristics between patients with and without DoCE (Table 1). In comparison of procedural characteristics at the lesion level, cases of left circumflex artery as a target lesion were more common, and reference vessel and stent diameter were smaller in the lesions with DoCE (Table 2). Post-stent OCT findings are shown in Table 3. The mean MSA by post-stent OCT in overall population was 6.3 ± 2.0 mm^2^. Volumetric stent expansion rate by adaptive reference lumen volume was 54.5 ± 16.3% in this cohort. The average reference lumen area (6.8 ± 1.9 mm^2^ vs 7.7 ± 2.5 mm^2^, *P* = 0.003) and MSA (5.6 ± 1.8 mm^2^ vs 6.3 ± 2.0 mm^2^, *P* = 0.014) were significantly smaller in lesions with than without DoCE. Relative stent expansion indexes such as conventional stent expansion (MSA/average reference lumen area), MSA by distal reference lumen area or mean stent expansion were comparable between lesions with and without DoCE. However, the degree of volumetric stent expansion which was represented as stent expansion by linear model was significantly higher in lesions with than without DoCE (Table 3).

**Table 1:** Patient characteristics.

	All patients (n = 1071)	Patients with DOCE (n = 44)	Patients without DOCE (n = 1027)	P value
Age (years)	61.3 ± 9.5	63.0 ± 10.2	61.2 ± 9.5	0.223
Males, n (%)	774 (72.3)	32 (72.7)	742 (72.2)	1.000
Body mass index (kg/m^2^)	24.7 ± 2.8	24.4 ± 3.1	24.7 ± 2.8	0.408
Comorbidities, n (%)				
Hypertension	631 (58.9)	27 (61.4)	604 (58.8)	0.827
Diabetes	306 (28.6)	10 (22.7)	296 (28.8)	0.480
Dyslipidemia	750 (70.0)	32 (72.7)	718(69.9)	0.814
Current smoker	258 (24.1)	11 (25.0)	247 (24.1)	1.000
Chronic kidney disease	9 (0.8)	2 (4.5)	7 (0.7)	0.057
Prior PCI	89 (8.3)	4 (9.1)	85 (8.3)	1.000
PCI indication, n (%)				0.162
Non-MI	893 (83.4)	33 (75.0)	860 (83.7)	
MI	178 (16.6)	11 (25.0)	167 (16.3)	
Laboratory data				
Hemoglobin (g/dl)	14.2 ± 1.6	14.1 ± 2.0	14.2 ± 1.5	0.650
White blood cell count (k/mm^3^)	7.45 ± 2.51	8.08 ± 2.73	7.42 ± 2.50	0.092
LDL cholesterol (mg/dl)	107.3 ± 35.1	114.1 ± 48.2	107.0 ± 34.5	0.432
HDL cholesterol (mg/dl)	44.5 ± 10.8	43.2 ± 11.5	44.5 ± 10.8	0.507
Creatinine (mg/dl)	0.9 ± 0.5	1.2 ± 1.5	0.9 ± 0.4	0.240
Coronary angiography				
Multivessel disease	515 (48.1)	24 (54.5)	491 (47.8)	0.381
Medication at discharge				
DAPT	1061 (99.1)	41 (93.2)	1020 (99.3)	0.054
Beta-blocker	701 (65.5)	29 (65.9)	672 (65.4)	1.000
ACE inhibitor/ARB	626 (58.5)	24 (54.5)	602 (58.6)	0.840
Statin	1027 (95.9)	40 (90.7)	987 (96.1)	0.182

*ACE* angiotensin-converting enzyme, *ARB* angiotensin II receptor blocker, *DAPT* dual antiplatelet therapy, *DoCE* device-oriented clinical end point, *HDL* high-density lipoprotein, *MI* myocardial infarction, *LDL* low-density lipoprotein, *PCI* percutaneous coronary intervention.

**Table 2:** Procedural characteristics.

	All lesions (n = 1123)	Lesions with DOCE (n = 44)	Lesions without DOCE (n = 1079)	P value
Treated lesion location				
Left main	18 (1.6)	0	18 (1.7)	0.802
Left anterior descending artery	657 (58.5)	22 (50.0)	635 (58.9)	0.312
Left circumflex artery	192 (17.1)	13 (29.5)	179 (16.6)	0.042
Right coronary artery	247 (22.0)	8 (18.2)	239 (22.2)	0.662
Ramus intermedius	9 (0.8)	1 (2.3)	8 (0.7)	0.799
Lesion features				0.253
A/B1	460 (41.0)	23 (52.3)	437 (40.5)	
B2/C	663 (59.0)	21 (47.7)	642 (59.5)	
Bifurcation	116 (10.3)	3 (6.8)	113 (10.5)	0.472
Preintervention QCA analysis				
Reference vessel diameter (mm)	3.1 ± 0.5	2.8 ± 0.4	3.1 ± 0.5	0.046
Lesion length (mm)	16.6 ± 4.9	16.8 ± 4.3	16.6 ± 5.0	0.892
Minimal lumen diameter (mm)	1.0 ± 0.6	0.9 ± 0.5	1.0 ± 0.6	0.396
% of stenosis	67.5 ± 14.4	69.1 ± 17.4	67.4 ± 14.2	0.680
Multiple stents, n (%)	26 (2.3)	0	26 (2.4)	0.596
Stent diameter (mm)	3.2 ± 0.4	3.1 ± 0.3	3.2 ± 0.4	0.015
Stent length (mm)	19.1 ± 6.3	19.2 ± 7.2	19.1 ± 6.3	0.916
Maximal inflation pressure (atm)	11.8 ± 3.2	12.4 ± 3.3	11.8 ± 3.2	0.252
Adjuvant balloon, n (%)	600 (53.4)	18 (40.9)	582 (53.9)	0.135

*DoCE* device-oriented clinical end point, *QCA* quantitative coronary angiography.

**Table 3:** Post-stent OCT findings.

	All lesions (n = 1123)	Lesions with DoCE (n = 44)	Lesions without DoCE (n = 1079)	P value
Average reference lumen area (mm^2^)	7.7 ± 2.5	6.8 ± 1.9	7.7 ± 2.5	0.003
MSA (mm^2^)	6.3 ± 2.0	5.6 ± 1.8	6.3 ± 2.0	0.014
MSA/average reference lumen area (%)	82.7 ± 13.0	82.0 ± 13.7	82.7 ± 13.0	0.715
MSA/distal reference lumen area (%)	94.0 ± 17.0	91.7 ± 16.8	94.1 ± 17.0	0.367
Mean stent expansion (%)	98.9 ± 16.2	102.5 ± 19.5	98.8 ± 16.0	0.222
SV/adaptive reference lumen volume (%)	54.5 ± 16.3	59.7 ± 17.3	54.3 ± 16.2	0.033

*DoCE* device-oriented clinical end point, *MSA* minimal stent area, *OCT* optical coherence tomography, *SV* stent volume.

### Association between stent expansion indices and outcomes

Among the several stent expansion indices, MSA (hazard ratio [HR], 0.80; 95% confidence interval [CI] 0.68‒0.94; *P* = 0.012) and stent expansion by linear model (HR 1.02; 95% CI 1.00‒1.04; *P* = 0.019) were found to be highly predictive of DoCE in univariate analysis (Table 4). Harrell’s C-index for the prediction of DoCE of stent expansion by linear model was found to be 0.603. The best cutting points of stent expansion by linear model to predict DoCE were determined to be ≥ 65.0% by a maximal chi-square method. The predictive performance of stent expansion by linear model based on this threshold was internally validated using 1000 bootstrapped resampling, and the resulting outcome of the bias-corrected Harrell’s C-index was 0.600, indicating similar reproduction of Harrell’s C-index in a validation step.

**Table 4:** Association between stent expansion indices and DoCE.

	HR (95% CI)	P value
MSA	0.80 (0.68–0.94)	0.008
MSA/average reference lumen area (%)	0.99 (0.97–1.02)	0.635
MSA/distal reference lumen area (%)	0.99 (0.97–1.01)	0.331
Mean stent expansion (%)	1.01 (1.00–1.03)	0.099
SV/adaptive reference lumen volume (%)	1.02 (1.00–1.04)	0.019

*CI* confidence interval, *DoCE* device-oriented clinical end point, *HR* hazard ratio, *MSA* minimal stent area, *SV* stent volume.

### Stent expansion criteria and outcomes

The risk of clinical outcomes was evaluated according to suboptimal stent expansion criteria such as MSA < 5.0 mm^2^, MSA/average reference lumen area < 90%, MSA/average reference lumen area < 80%, MSA/distal reference lumen area < 100%, MSA/distal reference lumen area < 90%, and stent expansion by linear model ≥ 65.0% (Table 5). After adjustment for confounders, MSA < 5.0 mm^2^ (HR 3.90; 95% CI 1.99‒7.65) (Table 5 and Fig. 3A), MSA by distal reference lumen area < 90% (HR, 2.16; 95% CI 1.12‒4.19) (Table 5 and Fig. 3B), and stent expansion by linear model ≥ 65.0% (HR, 1.95; 95% CI 1.03‒3.89) (Table 5 and Fig. 3C) were independently associated with increased risk of DoCE. Individual outcomes according to suboptimal stent expansion criteria are summarized in Supplemental Table 1. Summaries of representative cases of small MSA (< 5.0 mm^2^), MSA by distal reference lumen area < 90%, and stent expansion by linear model ≥ 65.0%, which resulted in adverse outcomes are presented in Fig. 4.

**Table 5:** The risk of DoCE according to OCT-defined stent expansion criteria.

Suboptimal criteria	Patients, n (%)		Adjusted HR (95% CI)	P value
	Suboptimal	Optimal		
MSA < 5.0 mm^2^	23/307 (7.5)	21/764 (2.7)	3.90 (1.99–7.65)	< 0.001
MSA/average reference lumen area < 90%	30/762 (3.9)	14/309 (4.5)	1.32 (0.62–2.81)	0.471
MSA/average reference lumen area < 80%	20/441 (4.5)	24 /630 (3.8)	1.33 (0.69–2.55)	0.390
MSA/distal reference lumen area < 100%	31/695 (4.5)	13/374 (3.5)	1.38 (0.67–2.86)	0.386
MSA/distal reference lumen area < 90%	24/422 (5.7)	20/647 (3.1)	2.16 (1.12–4.19)	0.022
SV/adaptive reference lumen volume ≥ 65.0%	17/256 (6.6)	27/813 (3.3)	1.95 (1.03–3.89)	0.042

Patient-level analysis was performed. Adjusted covariates include age, male sex, hypertension, diabetes, current smoker, chronic kidney disease, myocardial infarction, dual antiplatelet use at discharge, statin use at discharge, total stent length, mean stent diameter, and adjuvant ballooning.

*CI* confidence interval, *DoCE* device-oriented clinical end point, *HR* hazard ratio, *MSA* minimal stent area, *OCT* optical coherence tomography, *SV* stent volume.

### Association of stent expansion criteria and outcomes according to vessel size

As the MSA criterion may not be achievable in small vessels and may result in undersized or under expanded stents in large vessels, the association between stent expansion criteria and outcomes according to reference vessel size (small vessel, mean stent diameter < 3.0 mm; large vessel, mean stent dimeter ≥ 3.0 mm) (Supplemental Fig. 1) was tested. In small vessels, the achievement rate of MSA ≥ 5.0 mm^2^ was low (34 of 191, 17.8%), and the event rate according to the MSA criterion was similar. Although there was no statistical significance, a trend of higher DoCE rate in case of MSA/distal reference lumen area < 90% and stent expansion by linear model < 65.0% in small vessels, was observed. In large vessels, the impacts of MSA < 5.0 mm^2^ and stent expansion by linear model ≥ 65.0% on DoCE were consistent. Event rate of MSA/distal reference lumen area < 90% tended to be higher than that of ≥ 90%; however, statistical significance was not reached. On testing stent expansion by the linear model criterion in case of MSA ≥ 5.0 mm^2^ in large vessels (mean stent diameter ≥ 3.0 mm), stent expansion by linear model ≥ 65.0% was associated with increased risk of DoCE (Supplemental Fig. 2).

## Discussion

The present analysis based on a large population-based OCT registry demonstrated that (1) among several stent expansion indices, MSA was negatively correlated with the risk of DoCE, whereas, stent expansion by linear model which is the overall volumetric stent expansion concept was associated with higher incidence of DoCE; (2) suboptimal stent expansion with MSA < 5.0 mm^2^ and MSA by distal reference lumen area < 90% were independently associated with increased risk of DoCE; however, excessive volumetric stent expansion with stent expansion by linear model ≥ 65.0% was also a significant determinant of DoCE. Our results highlight that meticulous effort to alleviate stent underexpansion based on intravascular imaging findings is important to improve clinical outcomes. In addition, our finding suggests that, for the first time to our knowledge, not only stent underexpansion but also overall excessive stent expansion can worsen PCI outcome.

Although achievement of absolute stent expansion is limited by vessel size or a risk of stent size mismatch, the MSA has been reported to be an important predictor of future events in both intravascular ultrasound (IVUS) and OCT studies^2,5–7,19^. Data from CLI-OPCI (Centro per la Lotta contro l’Infarto–Optimisation of Percutaneous Coronary Intervention)^6^ and Massachusetts General Hospital OCT registries ^5^ identified MSA of 4.5 mm^2^ and 5.0 mm^2^ by OCT as thresholds for discriminating future events after implantation of all generation DES, respectively. The DOCTORS (Does Optical Coherence Tomography Optimize Results of Stenting) study reported an MSA > 5.44 mm^2^ can predict post-PCI fractional flow reserve (FFR) > 0.90; however, the number of participants using OCT-guided PCI was only 120^7^. In this analysis of Yonsei OCT registry data, absolute stent expansion of MSA > 4.5 mm^2^, > 5.0 mm^2^, and > 5.44 mm^2^ were achieved in 80.4%, 70.6% and 62.8% of the patients, respectively. The MSA threshold of 4.5 mm^2^ was too small for large vessels and considering that MSA ≥ 5.0 mm^2^ was achieved for only 17.8% of the small vessels treated with a mean stent diameter < 3.0 mm, achieving MSA > 5.44 mm^2^ is a very large threshold for those vessels. Therefore, the MSA threshold of 5.0 mm^2^ generally seems to be reasonable. Consistent with previous studies ^1,5,20^, MSA < 5.0 mm^2^ was an independent predictor of DoCE in our study.

With respect to relative stent expansion, a uniform criteria using the average or distal reference lumen area has not been established yet. Among several relative stent expansion criteria, achieving > 80% for the MSA/average reference lumen area is recommended by the latest expert consensus document ^4^, as achieving > 90% expansion was very challenging in previous studies, and DOCTORS study reported that the cut-off of > 79.4% expansion could predict post-stent FFR > 0.90^7^. However, this criterion is controversial as the MSA/average reference lumen area > 80% can result in a small MSA in small vessels^21^. Furthermore, in the analysis of pooled data of IVUS-XPL (Impact of Intravascular Ultrasound Guidance on the Outcomes of Xience Prime Stents in Long Lesions) and ULTIMATE (Intravascular Ultrasound Guided Drug Eluting Stents Implantation in All-Comers Coronary Lesions), neither an MSA/average reference lumen area > 90% nor > 80% could affect the three-year clinical outcomes^15^. Instead, MSA/distal reference lumen area > 90% was associated with improved three-year outcomes. Consistently, MSA/distal reference lumen area < 90% was found to be more predictive for long-term DoCE than MSA/average reference lumen area criteria in our OCT study. Recently, concept of volumetric stent expansion index using the H–K model described by Huo et al. ^22^, which takes vessel tapering into account, has been proposed; it shows a better correlation with one-year DoCE^9^. However, in an IVUS substudy from ADAPT-DES (Assessment of dual antiplatelet therapy with drug-eluting stents) including 1831 patients and 2140 lesions, minimum stent expansion by the H–K model did not show correlation with two-year outcomes, whereas, the MSA/vessel area at MSA site ratio in IVUS was superior to conventional expansion indexes in predicting adverse events^23^. A recent study reported that the smaller value of the OCT-derived stent expansion index which was calculated separately for each halves of stented segment using MSA/average reference lumen area was predictive of adverse clinical outcomes when the value was less than 0.85^24^. Unfortunately, these recent new indexes were not evaluated in this study; further research is needed for validation using both OCT and IVUS as the results are not consistent. Currently, cut-off > 90% for MSA/distal reference lumen area may be considered as a relative stent expansion target after DES implantation based on the consistent results from both IVUS and OCT studies. Considering that only 18% achieved MSA ≥ 5.0 mm^2^ in vessel size less than 3.0 mm, achieving MSA ≥ 5.0 mm^2^ is very important in larger vessels. Relative stent expansion with MSA/distal reference lumen area ≥ 90% may be the target in smaller vessels, which often have difficulty in achieving MSA ≥ 5.0 mm^2^ (Supplemental Fig. 1).

Previously, we demonstrated that a significant stent malapposition with total malapposition volume ≥ 7.0 mm^3^ is associated with future thrombotic events after PCI with DES^11^. Taken together, the results of previous and the present studies suggest that mitigating both stent underexpansion and severe malapposition is an important task for stent optimization even after new-generation DES implantation.

In the present study, we conducted a volumetric assessment based on the concept of overall and minimal stent expansion for lesions. A volumetric analysis may evaluate both the effect on the underexpansion area and the entire vessel wall expansion in which the stent is implanted. Although clinical PCI studies have shown that final lumen diameter or area after PCI is a major determinant of the rate of restenosis ^2,25^, some previous animal and human PCI studies have demonstrated that vessel wall injury by balloon overstretch or stenting during procedure can promote intimal hyperplasia^26–28^. Previous serial IVUS observational study showed that increase of total vascular area rather than lumen or plaque area was highly related to the magnitude of in-stent neointimal growth, suggesting that stretch or injury to the adventitia rather than intima has an effect on neointimal hyperplasia^28^. A previous porcine animal study demonstrated similar findings that adventitial myofibroblasts play a role in vascular lesion formation by proliferation, migration, and stimulation of growth factors after balloon overstretch injury to coronary arteries^29^. Therefore, there is a trade-off between gain of larger acute lumen area and the degree of vascular injury during PCI. However, the effect of excessive stent expansion during PCI on long-term clinical outcomes has not been sufficiently evaluated. A previous OCT study have demonstrated that the ratio of stent area at border to averaged lumen area in the stent edge segment was significantly greater in the stent edge restenosis group compared with the non-stent edge restenosis group^30^. Interestingly, in a similar context, the percentage of volumetric stent expansion by adaptive vessel volume was positively correlated with the risk of DoCE after PCI, and especially, stent volume/adaptive reference lumen volume ≥ 65.0% was independently associated with higher incidence of DoCE after adjustment for other confounders in our study. This finding suggests that it is necessary to cover minimum expansion area by dilatation and overall excessive dilatation of stent may lead to increased risk of adverse events.

Excessive volumetric stent expansion can occur as a result of post-stent overdilatation with adjuvant balloon. As stent underexpansion is recognized as a risk factor of stent failure, post-dilatation after stenting is often performed with an expectation of larger luminal gain. Post-dilatation is usually applied throughout the entire stented area; however, its clinical benefit after DES implantation is unclear. Hong et al. reported that post-dilatation was not associated with improved clinical outcomes at 12 months after long everolimus-eluting stent implantation^31^. In addition, Lee et al. recently demonstrated that angiography-guided post-dilatation did not improve clinical outcome in comparison to non-post-dilatation group (HR, 0.76; 95% CI 0.50‒1.15; P = 0.194), whereas IVUS-guided post-dilatation was associated with better long-term outcome (HR, 0.35; 95% CI 0.2‒0.56; P < 0.001)^32^. Routine post-dilatation throughout the stented area may lead to overall stent overexpansion and vascular injury. Both previous findings and our results implicate that post-stent intravascular imaging guided post-dilatation may be more effective than routine post-dilatation based on angiographic findings to achieve sufficient dilatation where the stent is suboptimaly expanded and reduce unnecessary stent overexpansion.

Based on post-stent OCT findings, post-dilatation may be needed only if there is a site of suboptimal stent expansion, such as MSA < 5.0 mm^2^ or MSA/distal reference lumen area < 90%. However, unnecessary or routine post-dilatation of all stented areas from proximal to distal, which can lead to excessive volumetric stent expansion, may result in increasing adverse events.

### Study limitations

We acknowledge several limitations related to our study. First, although the registry data were collected prospectively, this study was a non-randomised observation study with post-hoc analysis for clinical outcomes. Second, since OCT was performed based on operators’ discretion, our study might include some selection bias, owing to which our findings cannot be generalised. However, no specific indication and recommendation for stent optimization under OCT guidance allowed to investigate clinical outcomes according to varying stent expansion rate in real-world practice. Third, 308 of 1379 (22.3%) selected patients with adequate OCT images were excluded from this analysis due to clinical follow-up loss within one year, which may affect the results of the present study. Fourth, the volumetric concept of stent expansion of our study is novel; therefore, it has not been validated. Fifth, plaque characteristics such as attenuated plaque or calcific nodules may affect the stent expansion indexes. The influence of those plaque characteristics were not evaluated since the information regarding plaque morphologies or pre-procedural OCT images were missed. Sixth, although we have suggested the cut-off for volumetric stent expansion by linear model, it is difficult to apply these thresholds for guidance to avoid overstretching during stent implantation in real clinical practice; it requires further validation in future. Nevertheless, our data implicates for the first time that it is better to avoid overstretching the total stent area if there is no significant malapposition and the criteria beyond the underexpansion threshold is met. Seventh, the adaptive reference lumen volume was calculated using the proximal and distal reference lumen area without considering presence of side branches. Seventh, due to the nature of OCT, it was possible to analyse the lumen area; however, the evaluation of the total vessel area was not possible. Finally, understanding the effect of stent under- and over-expansion on sequential neointimal hyperplasia, stent coverage, and their clinical consequences will require future investigations with serial follow-up OCT studies.

## Conclusions

In conclusion, suboptimal stent expansion with MSA < 5.0 mm^2^ and MSA/distal reference lumen area < 90% as assessed by OCT was independently associated with DoCE after new-generation DES implantation. However, this study also demonstrated, for the first time that overall stent overexpansion defined as stent volume/adaptive reference lumen volume ≥ 65.0% is another predictor of DoCE. Therefore, OCT-guided selective stent optimization may be necessary; however, it should be considered to avoid unnecessary or overall routine post-dilatation leading to excessive stent overexpansion.

## Supplementary Information

Supplementary Information.

## Data Availability

The data that support the findings of this study are available from the corresponding author upon reasonable request.

## Abbreviations

DES: Drug-eluting stent
DoCE: Device-oriented clinical endpoints
FFR: Fractional flow reserve
IVUS: Intravascular ultrasound
MSA: Minimal stent area
OCT: Optical coherence tomography
PCI: Percutaneous coronary intervention
TLR: Target lesion revascularization
