# Supplementary material for: Stent expansion evaluated by optical coherence tomography and subsequent outcomes
Source: Sci Rep. 2023 Mar 7;13:3781. doi: 10.1038/s41598-023-30717-6 (PMC9992647; doi:10.1038/s41598-023-30717-6)
Supplement: Supplementary file 1 — Supplementary Information. [file 41598_2023_30717_MOESM1_ESM.docx]

**Supplementary Materials**

**Lee Bom, et al.** **Stent expansion evaluated by optical coherence tomography and subsequent outcomes**

**Contents**

**Supplemental methods**

**Supplementary Table 1.** The risk of individual outcomes according to OCT-defined stent expansion criteria

**Supplementary Figure 1.** Association of stent expansion criteria and outcomes according to vessel size (small vessel, mean stent diameter <3.0mm; large vessel, mean stent diameter ≥3.0mm)

**Supplementary Figure 2.** Outcomes stratified by minimal stent area and adaptive volumetric stent expansion criteria (≥65% versus <65%) in large vessels (mean stent diameter ≥3.0mm)

**Supplemental methods**

***Procedures***

Drug-eluting stents (DES) were chosen by operators’ discretion and included zotarolimus-eluting stents (Endeavor or Integrity, Medtronic, Minneapolis, Minnesota); everolimus-eluting stents (Xience, Abbott Vascular, Santa Clara, California; or Promus; Boston, Marlborough, MA); biolimus A9-eluting stents (BioMatrix, Biosensors International, Singapore; or Nobori, Terumo, Tokyo, Japan); sirolimus-eluting stents (Orsiro; Biotronik, Bülach, Switzerland); or novolimus-eluting (DESyne, Elixir Medical Corporation, Sunnyvale, CA). Stent implantation was performed according to current standard techniques and medical guidelines. Intravenous heparin was given at the start of the procedure (8000 to 10,000 IU bolus) to maintain an activated clotting time of 220 to 300 s. All patients were administered 300mg of aspirin plus either 300-600mg of clopidogrel, 180mg of ticagrelor or 10mg of prasugrel before procedure. After PCI, the patients were maintained 100mg of aspirin plus 75mg of clopidogrel, or 180mg of ticagrelor or 10mg of prasugrel. Details of the intervention, application of mechanical support, or concomitant medication, were left to the discretion of the operator.

**Supplementary Table 1. The risk of individual outcomes according to OCT-defined stent expansion criteria**

|  | Patients, n (%) | |  |  |
| --- | --- | --- | --- | --- |
| Suboptimal criteria | Suboptimal | Optimal | HR (95% CI) | P value |
| Cardiac death |  |  |  |  |
| MSA <5.0 mm^2^ | 7 / 307 (2.3) | 4 / 764 (0.5) | 4.65 (1.36-15.88) | 0.014 |
| MSA/average reference lumen area <90% | 9 / 762 (1.2) | 2 / 309 (0.6) | 1.87 (0.40-8.67) | 0.422 |
| MSA/average reference lumen area <80% | 8 / 441 (1.8) | 3 / 630 (0.5) | 3.81 (1.01-14.38) | 0.048 |
| MSA/distal reference lumen area <100% | 7 / 695 (1.0) | 4 / 374 (1.1) | 0.95 (0.28-3.25) | 0.938 |
| MSA/distal reference lumen area <90% | 6 /422 (1.4) | 5 / 647 (0.8) | 1.90 (0.58-6.23) | 0.289 |
| SV/adaptive reference lumen volume ≥65.0% | 5 / 256 (2.0) | 6 / 813 (0.7) | 2.81 (0.86-9.21) | 0.088 |
| Target vessel-related myocardial infarction |  |  |  |  |
| MSA <5.0 mm^2^ | 3 / 307 (1.0) | 4 / 764 (0.5) | 1.98 (0.44-8.85) | 0.372 |
| MSA/average reference lumen area <90% | 6 / 762 (0.8) | 1 / 309 (0.3) | 2.44 (0.29-20.29) | 0.408 |
| MSA/average reference lumen area <80% | 4 /441 (0.9) | 3 / 630 (0.5) | 1.88 (0.42-8.38) | 0.410 |
| MSA/distal reference lumen area <100% | 7 / 695 (1.0) | 0 / 374 (0) | - | - |
| MSA/distal reference lumen area <90% | 5 / 422 (1.2) | 2 / 647 (0.3) | 3.83 (0.74-19.75) | 0.108 |
| SV/adaptive reference lumen volume ≥65.0% | 0 / 256 (0) | 7 / 813 (0.9) | - | - |
| Stent thrombosis, definite |  |  |  |  |
| MSA <5.0 mm^2^ | 3 / 307 (1.0) | 3 / 764 (0.4) | 2.51 (0.51-12.44) | 0.260 |
| MSA/average reference lumen area <90% | 4 / 762 (0.5) | 2 / 309 (0.6) | 0.81 (0.15-4.43) | 0.809 |
| MSA/average reference lumen area <80% | 3 / 441 (0.7) | 3 / 630 (0.5) | 1.43 (0.29-7.07) | 0.663 |
| MSA/distal reference lumen area <100% | 4 / 695 (0.6) | 2 / 374 (0.5) | 1.07 (0.20-5.85) | 0.936 |
| MSA/distal reference lumen area <90% | 4 / 422 (0.9) | 2 / 647 (0.3) | 3.07 (0.56-16.76) | 0.195 |
| SV/adaptive reference lumen volume ≥65.0% | 1 / 256 (0.4) | 5 / 813 (0.6) | 0.64 (0.07-5.48) | 0.684 |
| Target lesion revascularization |  |  |  |  |
| MSA <5.0 mm^2^ | 17 / 307 (5.5) | 16 / 764 (2.1) | 2.78 (1.41-5.51) | 0.003 |
| MSA/average reference lumen area <90% | 21 / 762 (2.8) | 12 / 309 (3.9) | 0.72 (0.35-1.47) | 0.366 |
| MSA/average reference lumen area <80% | 13 / 441 (2.9) | 20 / 630 (3.2) | 0.92 (0.46-1.86) | 0.827 |
| MSA/distal reference lumen area <100% | 24 / 695 (3.5) | 9 / 374 (2.4) | 1.44 (0.67-3.09) | 0.355 |
| MSA/distal reference lumen area <90% | 17 / 422 (4.0) | 16 / 647 (2.5) | 1.67 (0.85-3.31) | 0.140 |
| SV/adaptive reference lumen volume ≥65.0% | 13 / 256 (5.1) | 20 / 813 (2.5) | 2.18 (1.08-4.37) | 0.029 |

Patient-level analysis was performed. CI, confidence interval; DoCE, device-oriented clinical end point; HR, hazard ratio; MSA, minimal stent area; OCT, optical coherence tomography; SV, stent volume


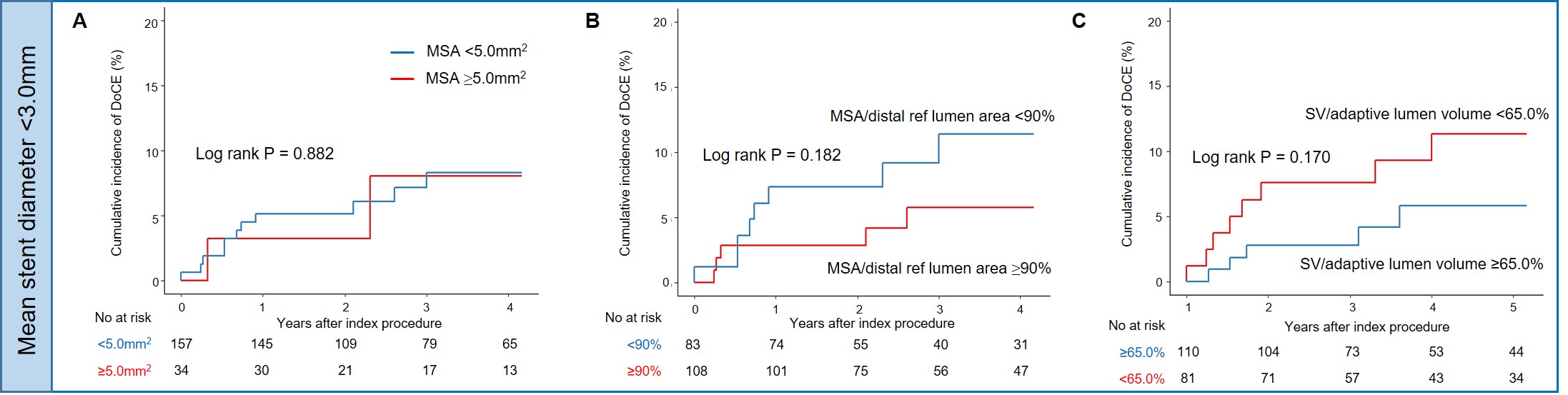


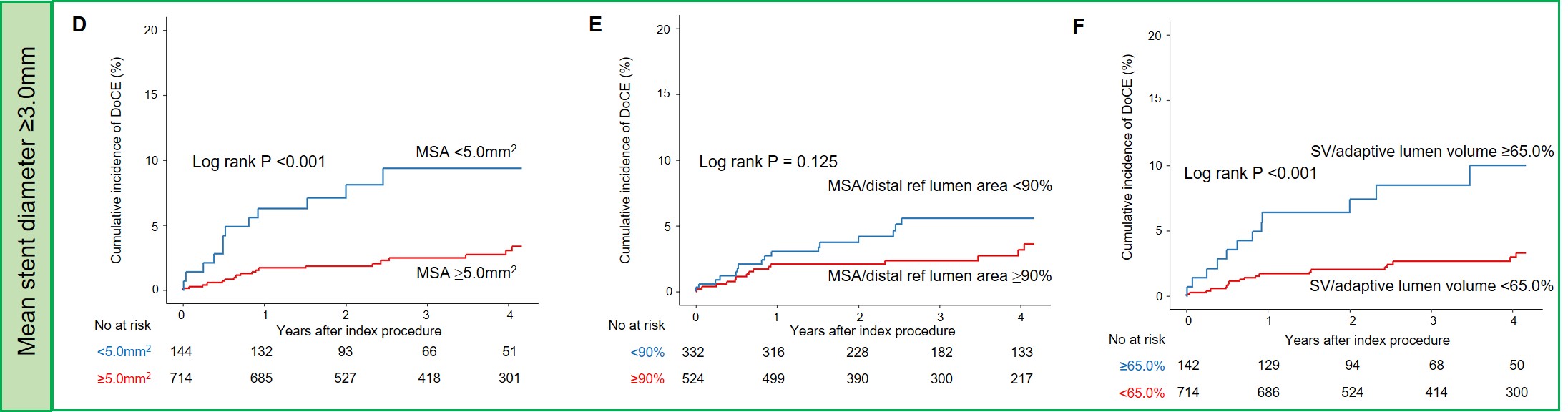


**Supplementary Figure 1. Association of stent expansion criteria and outcomes according to vessel size (small vessel, mean stent diameter <3.0mm; large vessel, mean stent diameter ≥3.0mm)**

DoCE, device-oriented clinical endpoints; MSA. minimal stent area; SV, stent volume


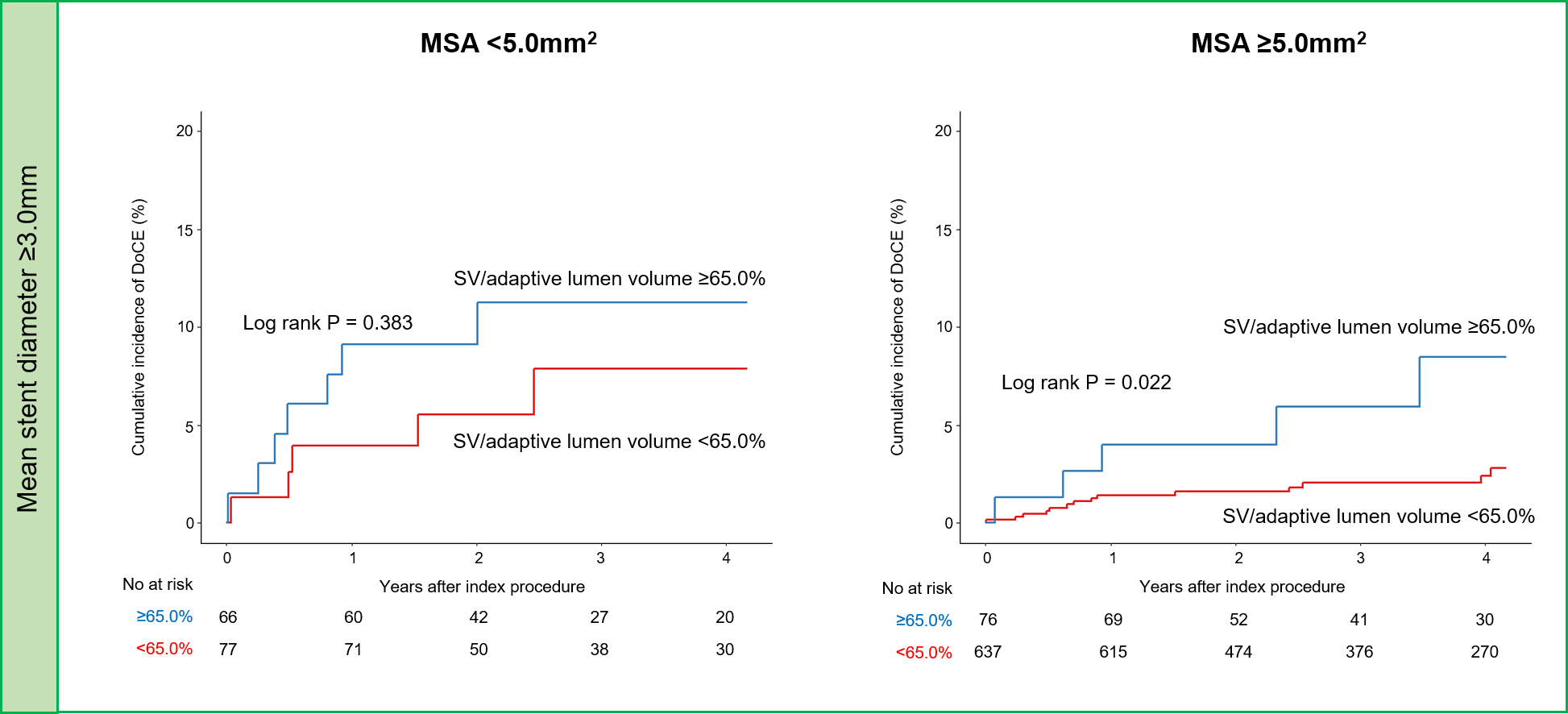


**Supplementary Figure 2. Outcomes stratified by minimal stent area and adaptive volumetric stent expansion criteria (≥65% versus <65%) in large vessels (mean stent diameter ≥3.0mm)**

DoCE, device-oriented clinical endpoints; MSA. minimal stent area; SV, stent volume
